# Framing the Health Workforce Agenda Beyond Economic Growth

**DOI:** 10.15171/ijhpm.2018.45

**Published:** 2018-05-16

**Authors:** Remco van de Pas, Linda Mans, Marielle Bemelmans, Anja Krumeich

**Affiliations:** ^1^Maastricht Centre for Global Health, Maastricht University, Maastricht, The Netherlands.; ^2^Institute of Tropical Medicine, Antwerp, Belgium.; ^3^Wemos Foundation, Amsterdam, The Netherlands.; ^4^Department of Health Ethics and Society, Faculty of Health Medicine and Life Sciences, Maastricht University, Maastricht, The Netherlands.

**Keywords:** Health Employment, Economic Growth, Labor Markets, Global Health Framing, Fiscal Space

## Abstract

The fourth Global Forum on Human Resources (HRH) for Health was held in Ireland November 2017. Its Dublin declaration mentions that strategic investments in the health workforce could contribute to sustainable and inclusive growth and are an imperative to shared prosperity. What is remarkable about the investment frame for health workforce development is that there is little debate about the type of economic development to be pursued. This article provides three cautionary considerations and argues that, in the longer term, a perspective beyond the dominant economic frame is required to further equitable development of the global health workforce. The first argument includes the notion that the growth that is triggered may not be as inclusive as proponents say it is. Secondly, there are considerable questions on the possibility of expanding fiscal space in low-income countries for public goods such as health services and the sustainability of the resulting economic growth. Thirdly, there is a growing consideration that economic growth solely expressed as increasing gross domestic product (GDP) might have intrinsic problems in advancing sustainable development outcomes. Economic development goals are a useful approach to guiding health workforce policies and health employment but this depends very much on the context. Alternative development models and policy options, such as a Job Guarantee scheme, need to be assessed, deliberated and tested. This would meet considerable political challenges but a narrow single story and frame of economic development is to be rejected.

## Health Employment and Economic Growth

The Fourth Global Forum on Human Resources for Health (HRH), held in Ireland November 2017, had the aim of furthering a bold economic case for investing in the health and social workforce, and intensifying inter-sectoral coordination. The Dublin declaration builds on the report of the UN Secretary-General’s High-level Commission on Health Employment and Economic Growth (UNHEEG) and its benefits across the Sustainable Development Goals (SDGs).^1,2^ The declaration also mentions that strategic investments in the health workforce could contribute to sustainable and inclusive growth and are an imperative to shared prosperity. Over the last few years, the World Health Organization (WHO) has made economic growth the dominant, but not exclusive, frame for health workforce development. In its publications WHO explicitly mentions the need to frame the health workforce agenda in a way that generates political will for health workforce development.^3,4^ Social theory provides the insight that similar issues can be framed in different ways by different actors.^5^ Framing analysis and its relevance for global health governance and policy-making has become more prominent.^6^ The framing of global health challenges has important power implications for the determination of policies and action, and therefore on the solutions that are proposed for dealing with a problem.^5^ Nearly a century ago, in his thinking on ‘linguistic hegemony’ or ‘cultural hegemony,’ Gramsci already provided the analysis that “*In a vital sense language is politics, for it affects the way people think about power.”*^7^ Lipmann, around the same time, introduced the term ‘manufacturing consent’ as a possibility to shape and manipulate the public opinion in democratic societies.^8^

What is remarkable about the investment case as a frame for health workforce development is that there is little debate about the type of economic development to be pursued. Rather, ‘inclusive growth’ as the outcome of health workforce investments is considered a given. This article provides three cautionary considerations of this principle and argues that in the longer term a perspective beyond the dominant economic frame is required to further equitable development of the global health workforce.

## The Health Labor Market and Fiscal Space

The WHO has made labor market analysis the central framework for assessing health workforce requirements both at national and global levels. It uses supply-, need- and demand- models to provide scenarios on how the workforce will likely develop over the following years.^9^ Over the years, the World Bank (WB) has become more engaged in health workforce development. Guided by its focus on employability, poverty eradication and shared prosperity the WB has recognized that the health services sector provides a considerable economic growth potential contrary to ‘traditional’ industrial and extractive sectors. The WB has conceptually paved the way to assess health services from a labor market and fiscal perspective as a strategy for economic development in low- and middle-income countries (LMIC).^10,11^ Both in the domain of health workforce financing as well as health financing strategies such as universal health coverage (UHC) the WB has started to collaborate closely with the WHO. This does not only include cooperation and exchange at the technical level but also joint leadership and global commitment for UHC.^12^

The labor market framework provides the insight that in LMIC a major problem is not merely the lack of an available skilled health workforce but also the insufficient (economic) demand to finance health sector employment, thereby emphasizing the need to invest in jobs. Evidence also suggests that health labor markets are not intrinsically well-functioning. In order to reach a ‘market-cleaning equilibrium’ health labor markets require regulatory or institutional interventions to achieve socially desirable and economically efficient outcomes (eg, universal access to a health worker’s services via incentives to having the health worker retained for employment in rural areas).^13^ The WHO provides a conceptual overview of how investing in health systems can lead, via six pathways, to economic growth. The authors hereby mention that the concepts ‘efficiency’ and ‘growth’ are interlinked and provide two arguments for why growth is relevant for societies; first, by *“producing more benefits, in terms of income, consumption, investment, production, and other forms of (mainly) market-valued benefits.”* Secondly, growth requires government action to correct market failures (inefficiency) such as negative externalities and to provide public goods (eg, education and health care).^14^ The question is then, how to pay the wage bill for the additional health workforce required in order to attain the SDGs, and how to secure fiscal (public) and financial (public and private) space? An analysis shows that conditional on “*current trends of economic development and population growth”* there are challenges to securing the wage bill in a (small) number of countries (optimistically, as few as 4-16 countries; less optimistically, as many as 69 countries). The authors conclude “*that the number of countries requiring sustained development assistance for wage bills from donor nations is likely to be limited, possibly to as few as 20–30 countries or even fewer.”*^15^

In the analysis above the underlying notion is that economic growth, properly regulated by public authorities, is required to meet the Sustainable Development Agenda and its health related goals. In essence the SDGs are *“one gigantic global green version of Roosevelt’s New Deal agenda”* to advance socio-economic and ecological goals.^16^ However, Labonté notes that there is a contradiction at the heart of the SDGs that builds on an implicit assumption that the same economic rules that have created an increasingly unequal and unsustainable world can somehow engineer the reverse.^16^

## Alternative Analyses on Economic Development Needs

Three critical remarks can be made about the concept of (inclusive) economic growth being the desirable outcome of investments in health employment. The first considers the notion that the growth that is triggered may not be as inclusive as proponents say it is. While there is evidence that regulated economic growth might improve equitable access to health services, this attribution is not self-evident for health inequalities in principle. A range of countries in several regions of the world have used economic growth to enhance access to health professionals and sustainable health employment.^17-19^ At the same time income and wealth inequalities in many countries, although not necessarily between countries, have been growing over the last decades. Social policies as a way of public redistribution, such as social protection as indicated in one of the pathways by WHO, are a possible instrument for reducing income inequalities which would in turn lead to a reduction in health inequalities.^20^ However, this is not sufficient. Milanovic, by pointing out the growing disconnect between labor and capital, analyzes this ‘new capitalism’ as a major reason for the growth in global inequalities andargues for long-term equalization of capital ownership and education.^21^ Rather than focusing on economic growth via mere investment in labor (such as in the health workforce), a reduction of income inequalities via high-inheritance taxes, corporate tax policies and broader ownership of assets (by the poor and middle-class), as well as equalizing meaningful access to education, are policy recommendations for effectively reducing inequalities. The assets that would become available could then be re-invested in health employment and building a sustainable workforce. Milanovic makes the case that economic growth is still needed in poor countries. However, to make it ecologically sustainable, restraints on growth should be imposed on the rich countries.^21^ SDG goal 10, on reducing global inequality, is disappointing as only target 10.1 has as its aim to *“progressively achieve income growth for the bottom 40% of the population.”* Big drivers of poverty and global inequalities accumulated through private wealth accumulation are neglected by the SDGs and remain unaccounted for.^22^

Secondly, there are considerable questions by scholars on the possibility of expanding fiscal space for public goods in low-income countries and the sustainability of the resulting economic growth. Assessing fiscal space for financing health systems has gained momentum by health economists, but their main focus has been on increasing domestic revenues in line with recommendations by the Addis Ababa Action Agenda on financing for development.^23^ Rodrik, however, has clarified the tension between national democratic decision space and global markets as the ‘political trilemma of the world economy.’ In this trilemma there are basically three options; *restrict democracy* in the interest of minimizing international transaction costs (eg, labor wages); *limit globalization* in the hope of building democratic legitimacy at home; or to *globalize democracy* at the cost of national sovereignty. The ‘trilemma’ exists in the challenge that at most two out of these three options can function together. Too often the reality of sovereign nation states functioning in a hyper-globalized order is them being locked in a ‘Golden Straitjacket.’^24^ In this model, national, democratic, economic and fiscal policy space and its governance is inevitably restricted. The other possibly attractive options of limiting globalization by rethinking trade and investment agreements in order to expand democratic decision-making or to globalize democratic governance along with markets have so far had too much resistance from both old and new major state powers.^24^ The limitations of fiscal flexibility are outlined in an analysis on the impact of International Monetary Fund (IMF) conditionality (1995-2014) on government health expenditure in 16 African nations. Despite the rhetoric that in recent years the IMF has started to promote social protection policies and health systems strengthening, the evidence reveals that, under direct IMF tutelage, these countries have had limited policy space and considerably underfunded their health systems.^25^ For instance in Malawi 60% of the wage bill for the required staff establishment to meet essential health services is not funded [Clinton Health Access Initiative & Ministry of Health, Unpublished data, 2016]. Albeit health professional staff graduating in significant numbers, often with the scholarship support of donors, there has also been a freeze on the recruitment of staff. This has followed IMF recommendations to the government that a key priority in the short term is to restore macro-economic stability and that *“an appropriately tight fiscal policy is needed.”*^26^ Despite assumptions of continued economic growth, characterized by a divergence of paths between countries, Africa’s economies have seen a slowdown over the last couple of years.^27^ Health policies and its financing must incorporate the realities of non-linear economic growth and potential economic contraction. In the face of economic crisis, countercyclical measures should be brought in to mitigate its effects and provide social protection for low-income and vulnerable populations.^27^ In times of economic volatility, rather than leaving the onus of health employment financing in LMIC a sole domestic responsibility, it would be fairer to develop a coherent global framework for health financing based on shared responsibility principles. Such a framework is built on 4 principles (a global pact); domestic financing, joint financing of global public goods, external financing for national health systems, and a global agreement and accountability mechanism.^28^

Thirdly, and truly paradigmatically different, is the slow but growing consideration that economic growth solely expressed as increasing gross domestic product (GDP) might have intrinsic problems in advancing sustainable development outcomes. Woodward has calculated that under current ‘pro-poor’ economic development models it would take over 200 years to attain the eradication of poverty (measured at US$5 per person per day as poverty baseline). To do so, global GDP would have to increase to 175 times its present size. “*There is simply no way this can be achieved without triggering truly catastrophic climate change.”* It basically implies that we should shift our attention from global economic growth to the (re-)distribution of the benefits of global production and consumption.^29^ This principle is in essence also put forward by Raworth, who in her thinking on circular economics puts forward a model, the Doughnut, of social foundations and planetary boundaries (our ‘ecological ceiling’).^30^ She urges us to move from being growth addicted to being growth agnostic, and argues that economies should become distributive by design. This implies that investments in public goods, such as health employment, would be decoupled from economic growth and be achieved by tax justice and wealth redistribution, as outlined by Milanovic above. However, he considers such policy reforms not (yet) politically feasible in current times.^31^ In the circular economy, health employees would ideally work for public, democratic, accountable institutions or member/employee owned companies that would have a distributive enterprise design instead of a profit-oriented shareholders model.^30^ Stiglitz and Sen have put it very clearly; GDP is not a good measure of economic performance; it is not a good measure of well-being; it is a mismeasurement of life.^32^

Furthermore, the Degrowth economic paradigm and its movement are slowly gaining momentum. It postulates that all countries have a common but differentiated responsibility to fulfilling basic development goals. This would imply that poor countries may grow their economies until at least 2025, while richer countries downscale production and consumption by around 6% per year. This would allow poorer countries to use up a disproportionate share of the global carbon budget for socio-ecological development, for example by investing in health employment.^33^ The chairperson of the commission of the African Union has concurred as follows: *“African Youth represent more than 60% of the population in the continent. Without a heavy investment in this youth, its education, training, employment, and intellectual capacity…Africa does not have a future.”*^33^ He then continues as follows; *“The question of emigration, especially to Europe, arises in tragic terms. This is our common challenge. Our shared responsibilities here are excruciating; they challenge us in the depth of our consciences.”*^34^ In line with this plea, Milanovic and Rodrik both argue for a new deal on labor mobility; making the case for international agreements on facilitating temporary work visa programs including for labor mobility in the health services.^21,35^

## The Relevance of Economic Growth and Other Useful Frames

To be clear, we do not argue that the economic growth frame should be left unconsidered when reflecting on how to develop the health workforce and generate investments for health employment. A health labor market and fiscal space assessment can help make the right policy choices. The global strategy on HRH asserts that domestic resources for HRH should be supported by appropriate macroeconomic policies at national and global levels and that, at least under certain circumstances, *“countries will require overseas development assistance for a few more decades to ensure adequate fiscal space.”*^36^ Sustainable and inclusive economic growth in low-income countries is something to strive for. This is to be accompanied by progressive corporate tax policies, tackling illegitimate capital flight and closing down tax havens, as well as redistribution of the assets resulting from economic growth into social goods such as health services. Moreover the gender balance of health employment is also of relevance. Women constitute 60%-70% of the health workforce in most countries. Targeted investment in this labor group would contribute to addressing gender inequality at the workplace, with potential impacts in the household and in society in general.^37^

Nevertheless, the WHO and other key actors in health workforce policy must be encouraged to recognize, research, deliberate and test alternative frames, guiding health workforce development, and the different corresponding political pathways to change.^6^ When these actors claim ‘inclusive economic growth’ as the outcome of health workforce investments, they do so referring to SDG 1 (poverty elimination), SDG 3 (good health and well-being), SDG 4 (quality education), SDG 5 (gender equality) and SDG 8 (decent work and economic growth). However, the social determinants of both human health and environmental degradation should not be neglected.^38^ The security frame has often been invoked since the Ebola Outbreak of 2014-2015 in West-Africa. A skilled workforce is required to generate the capacities for global health security.^39^ Other policy options have somehow been neglected in the health workforce governance ‘discourse.’ For instance, the notion of the health workforce being a requirement for delivering Global Public Goods for Health (GPGH) has not been mentioned by the UNHEEG report. Functioning health systems can be considered an ‘access’ good for GPGH and presents a strong case for the provision of free health services at the national level, and for external subsidies needed to achieve this.^40^ Also, from a health equity perspective, values (frames) such as ‘health and human development,’ ‘health as a human right’ and ‘health and global justice’ are to be considered.^6^ From a development angle one could build on the health capability approach, and the implicit health systems and providers responsibility to pursuing this.^41^ Although there is reference by the UNHEEG report on the International Labor Organization’s recommendation 202 to the right to social protection, and gender equality, this is mostly applied to the social security rights of health workers themselves.^42^ A decade ago more attention was given to the human Right to Health and how it contributes to health workforce development.^43^

## A Job Guarantee Scheme for the Health Services Sector

Interestingly, more labor proposals are increasingly returning to a social policy framework that was popularized during and shortly after World War II; guaranteeing full employment as a strategy to realize macro-economic, redistributive and collective outcomes.^44^ The late Tony Atkinson, the godfather of inequality research, promoted a job guarantee scheme in his Magnus Opus; ‘Inequality, What can be done?’^45^ An elaborate proposal on the Job Guarantee, a public option for jobs, has recently been published. *“It is a permanent, federally funded, and locally administered program that supplies voluntary employment opportunities on demand for all who are ready and willing to work at a living wage.”*^46^ Future research is required to see if and how full employment schemes can be implemented and financed in the health care sector, assess its broader impact on socio-economic outcomes, and gauge the policy space that is possible in high-income counties as well as LMIC to pursue such social strategies.

Unfortunately, the human rights approach to health has largely been left out of the Sustainable Development Agenda.^47^ A global justice (shared responsibility) approach to health systems development and health employment, within ecological limits, could be materialized by effectuating mechanisms such as a coherent global framework for health financing, a Job Guarantee scheme or applying Raworth’s Doughnut model on circular economics to health systems development.^30,38^

## Conclusion: Framing and Differentiating the Health Workforce Agenda

In conclusion, economic development goals are a useful approach to guiding health workforce policies and health employment but this depends very much on the context. It does call for sustainable and inclusive economic growth in LMIC, and degrowth and delinking health employment from economic demand in countries beyond a certain income level. Low-income countries struggling to address health challenges still need sustained international support and targeted measures in order to address underlying inequities in the global health workforce distribution.^48^ This also requires the assessment, deliberation and testing of alternative development models and policy options, such as the Job Guarantee scheme. We realize that it would meet considerable political challenges but a narrow single story, a frame, of economic development is to be rejected. ‘*The future is fertile and rich with possibility; we need only have the courage to invent it.’*^29^

## Acknowledgements

We acknowledge the kind contributions of Clara Affun and Virginie Bakeroot in commenting and editing the manuscript.

## Ethical issues

Not applicable.

## Competing interests

Authors declare that they have no competing interests.

## Authors’ contributions

RP drafted a first version of the comment. LM, MB, and AK contributed with additional inputs. All authors agree with the final manuscript.

## Authors’ affiliations

^1^Maastricht Centre for Global Health, Maastricht University, Maastricht, The Netherlands. ^2^Institute of Tropical Medicine, Antwerp, Belgium. ^3^Wemos Foundation, Amsterdam, The Netherlands. ^4^Department of Health Ethics and Society, Faculty of Health Medicine and Life Sciences, Maastricht University, Maastricht, The Netherlands.

## References

[1] Fourth Global Forum on Human Resources for Health. Dublin Declaration on Human Resources for Health: Building the Health Workforce of the future. World Health Organization; 2017.

[2] Working for health and growth: investing in the health workforce. Report of the High-Level Commission on Health Employment and Economic Growth. World Health Organization; 2016.

[3] Cometto G, Boerma T, Campbell J, Dare L, Evans T (2013). The Third Global Forum: framing the health workforce agenda for universal health coverage. Lancet Glob Health.

[4] Framing the health workforce agenda for the Sustainable Development Goals: biennium report 2016–2017 — WHO Health Workforce. Geneva: World Health Organization; 2017.

[5] Koon AD, Hawkins B, Mayhew SH (2016). Framing and the health policy process: a scoping review. Health Policy Plan.

[6] McNeill D, Ottersen OP (2015). Global Governance for Health: how to motivate political change?. Public Health.

[7] Boothman D. The sources for Gramsci’s concept of hegemony. In: Green M, ed. Rethinking Gramsci. New York: Routledge; 2017:55-67.

[8] Lippmann W. Public Opinion. New York: Harcourt; 1922.

[9] Cometto G, Scheffler R, Liu J, et al. Health workforce needs, demand and shortages to 2030. In: Buchan J, Dhillon IS, Campbell J, eds. In Health Employment and Economic Growth: An Evidence Base. Geneva: World Health Organization; 2017:3-26.

[10] Vujicic M, Ohiri K, Sparkes S. Working in health: financing and managing the public sector health workforce. Washington: The World Bank; 2009.

[11] Soucat A, Scheffler R, Ghebreyesus TA. The labor market for health workers in Africa: a new look at the crisis. Washington: The World Bank; 2013.

[12] Ghebreyesus TA. Towards Universal Health Coverage: Tackling the Health Financing Crisis to End Poverty. Washington: The World Bank; 2018.

[13] McPake B, Maeda A, Araujo EC, Lemiere C, El Maghraby A, Cometto G (2013). Why do health labour market forces matter?. Bull World Health Organ.

[14] Lauer J, Soucat A, Araujo E, Weakliam D. Pathways: the health system, health employment, and economic growth. In: Buchan J, Dhillon IS, Campbell J, eds. In Health Employment and Economic Growth: An Evidence Base. Geneva: World Health Organization; 2017:174.

[15] Lauer J, Soucat A, Araújo E, et al. Paying for needed health workers for the SDGs: An analysis of fiscal and financial space. In: Buchan J, Dhillon IS, Campbell J, eds. In Health Employment and Economic Growth: An Evidence Base. Geneva: World Health Organization; 2017:236.

[16] Labonte R (2016). Health promotion in an age of normative equity and rampant inequality. Int J Health Policy Manag.

[17] Dal Poz MR, Sepulveda HR, Costa Couto MH (2015). Assessment of human resources for health programme implementation in 15 Latin American and Caribbean countries. Hum Resour Health.

[18] Dussault G, Badr E, Haroen H (2016). Follow-up on commitments at the Third Global Forum on Human Resources for Health: Indonesia, Sudan, Tanzania: “A commitment is a promise, a promise is a debt”. Hum Resour Health.

[19] van de Pas R, Veenstra A, Gulati D, Van Damme W, Cometto G (2017). Tracing the policy implementation of commitments made by national governments and other entities at the Third Global Forum on Human Resources for Health. BMJ Glob Health.

[20] Jutz R (2015). The role of income inequality and social policies on income-related health inequalities in Europe. Int J Equity Health.

[21] Milanovic B. What Next? Ten Short Reflections on the Future of Income Inequality and Globalization. in Global inequality. Harvard University Press; 2016:212-230.

[22] Hickel J. Five reasons to think twice about the UN’s Sustainable Development Goals. LSE Blog. 2015. http://blogs.lse.ac.uk/africaatlse/2015/09/23/five-reasons-to-think-twice-about-the-uns-sustainable-development-goals/.

[23] Barroy H, Kutzin J, Tandon A (2018). Assessing Fiscal Space for Health in the SDG Era: A Different Story. Health Systems & Reform.

[24] Rodrik D. The Globalization Paradox: Why Global Markets, States, and Democracy Can’t Coexist. Oxford University Press; 2011.

[25] Stubbs T, Kentikelenis A, Stuckler D, McKee M, King L (2017). The impact of IMF conditionality on government health expenditure: A cross-national analysis of 16 West African nations. Soc Sci Med.

[26] IMF Country Report Malawi No 15/345. https://www.imf.org/external/pubs/ft/scr/2015/cr15345.pdf. Published 2015.

[27] Russo G, Bloom G, McCoy D (2017). Universal health coverage, economic slowdown and system resilience: Africa’s policy dilemma. BMJ Glob Health.

[28] Ottersen T, Elovainio R, Evans DB (2017). Towards a coherent global framework for health financing: recommendations and recent developments. Health Econ Policy Law.

[29] Woodward D (2015). Incrementum ad absurdum: global growth, inequality and poverty eradication in a carbon-constrained world. World Econ Rev.

[30] Raworth K. Doughnut Economics: Seven Ways to Think Like a 21st-Century Economist. Chelsea Green Publishing; 2017.

[31] Milanovic B. The illusion of “degrowth” in a poor and unequal world. Global Inequality Blog. 2017. http://glineq.blogspot.nl/2017/11/the-illusion-of-degrowth-in-poor-and.html?m=1.

[32] Stiglitz JE, Sen A, Fitoussi JP. Mismeasuring Our Lives: Why GDP Doesn’t Add Up. The New Press; 2010.

[33] Hickel J. The Divide: A Brief Guide to Global Inequality and its Solutions. London: William Heinemann; 2017.

[34] Faki Mahamat M. Speech of the chairperson of the commission of the African Union. 5th African Union- European Union summit. Abidjan, Cote D’Ivoire: 2017. https://au.int/sites/default/files/speeches/33424-sp-speechnof_moussa_faki.pdf.

[35] Rodrik D. Globalisation: New Deal on Labour Mobility. Social Europe; 2018.

[36] World Health Organization. Global strategy on human resources for health: workforce 2030. Geneva: WHO; 2016.

[37] Langer A, Meleis A, Knaul FM (2015). Women and Health: the key for sustainable development. Lancet.

[38] McCoy D (2017). Critical global health: responding to poverty, inequality and climate change; Comment on “Politics, power, poverty and global health: systems and frames”. Int J Health Policy Manag.

[39] The Lancet (2016). No health workforce, no global health security. Lancet.

[40] Smith RD, Woodward D. Global Public Goods for Health: Use and Limitations. Trade, foreign policy, diplomacy and health. World Health Organization; 2003.

[41] Ruger JP (2010). Health capability: conceptualization and operationalization. Am J Public Health.

[42] International Labour Organization. Recommendation concerning National Floors of Social Protection. International Labour Organization; 2012:202.

[43] Health Workforce Advocacy Initiative. Incorporating the right to Health into Health Workforce Plans: Key considerations. Physicians for Human Rights; 2009. https://s3.amazonaws.com/PHR_other/incorporating-right-to-health.pdf.

[44] Klosse S, Muysken J (2016). Curbing the Labor Market Divide by fostering Inclusive Labor Markets through a Job Guarantee Scheme. Psychosociological Issues in Human Resource Management.

[45] Atkinson AB. Inequality, what can be done? Harvard University Press; 2015:133-154.

[46] Tcherneva PR. The Job Guarantee: Design, Jobs, and Implementation. Levy Economics Institute; 2018. https://econpapers.repec.org/paper/levwrkpap/wp_5f902.htm.

[47] Van de Pas R, Hill PS, Hammonds R (2017). Global health governance in the sustainable development goals: Is it grounded in the right to health?. Global Chall.

[48] Bemelmans M, Philips M. New plan to tackle the global shortage of health workers fails to address economic constraints. The BMJ Opinion. July 24, 2017. https://blogs.bmj.com/bmj/2017/07/24/new-action-plan-to-address-the-global-shortage-of-health-workers-fails-to-address-economic-constraints-to-its-implementation/.

